# Understanding Parental Decision-Making and Determinants of COVID-19 Vaccination for Children in Vietnam: A Cross-Sectional Online Survey

**DOI:** 10.3390/vaccines12111266

**Published:** 2024-11-08

**Authors:** Hien T. Nguyen, Khanh C. Nguyen, Thai Q. Pham, Hieu T. Nguyen, Anh Hoang, Trang T. Vu, Huyen T. Nguyen, Nghia D. Ngu, Florian Vogt

**Affiliations:** 1 National Centre for Epidemiology and Population Health, College of Health and Medicine, Australian National University, Canberra, ACT 2601, Australia; vuthutrang.hmu@gmail.com (T.T.V.); huyen.nguyen@anu.edu.au (H.T.N.); florian.vogt@anu.edu.au (F.V.); 2 Department of Communicable Disease Control, National Institute of Hygiene and Epidemiology, Hanoi 100000, Vietnam; nck@nihe.org.vn (K.C.N.); pqt@nihe.org.vn (T.Q.P.); ndn@nihe.org.vn (N.D.N.); 3 Field Epidemiology Training Program, National Institute of Hygiene and Epidemiology, Hanoi 100000, Vietnam; 4 Research Methodology and Biostatistics Department, School of Preventive Medicine and Public Health, Hanoi Medical University, Hanoi 100000, Vietnam; 5 Thai Nguyen Center for Disease Control and Prevention, Thai Nguyen 250000, Vietnam; daonguyenngocdiep@gmail.com (H.T.N.); anh197@gmail.com (A.H.); 6 The Kirby Institute, University of New South Wales, Sydney, NSW 2052, Australia

**Keywords:** COVID-19 vaccination, vaccine hesitancy, vaccine acceptance, parents, child health, health belief model

## Abstract

Background/Objectives: In Vietnam, COVID-19 vaccination campaigns for children have encountered numerous challenges due to acceptance issues among parents. This study aimed to assess parental decision-making and identify factors influencing their decision to vaccinate their children against COVID-19. Methods: This was a cross-sectional online survey conducted between April and May 2023 among parents of children aged 6–17 years enrolled in urban and rural schools in Thai Nguyen province, Vietnam. Data on parental and child demographics, vaccination decision-making, COVID-19 experiences, and health beliefs based on the Health Belief Model were collected and analyzed, using univariate and multivariable multinomial regression analyses. Results: Among 4235 respondents (median age 41 years, 80.4% female), 81.3% had accepted all vaccine doses for their children, 9.7% had accepted some doses, 4.6% had rejected all doses, and 4.5% had not vaccinated their children for reasons unrelated to vaccine acceptance. Factors influencing parental decision-making included parental age, educational status, area of residence, health beliefs, prior experience with COVID-19 vaccination, and their child’s age and health status. Conclusions: We found overall high levels of parental acceptance for COVID-19 vaccination for children in Thai Nguyen province. To enhance COVID-19 vaccination acceptance, targeted communication strategies should focus on younger parents, those living in urban areas, parents with higher educational levels, and those with children who are younger or have underlying medical conditions. Trusted sources such as healthcare workers, teachers, and official health websites are essential for disseminating accurate information and fostering trust in vaccination programs.

## 1. Introduction

The global response to the COVID-19 pandemic has highlighted the paramount importance of vaccination as a fundamental public health tool [1]. While the initial focus was on vaccinating adults, attention has now shifted towards the vaccination of children. Children, constituting a significant segment of the population, are essential targets for vaccination efforts. Their vaccination not only safeguards their own well-being but also plays a pivotal role in reducing severity of illness in the population [2].

In this context, parental decision-making emerges as a critical determinant of the success of childhood vaccination programs, including those against COVID-19. Parents serve as primary decision-makers for their children’s health, and their choices are influenced by an array of determinants. These determinants encompass a wide spectrum, including perceived vaccine risks and benefits, healthcare access, cultural beliefs, and societal influences [3].

Located in South-East Asia, Vietnam is a lower-middle-income country with more than 27 million children, accounting for 28% of the total population [4]. In Vietnam, the Ministry of Health has granted approval for the administration of two COVID-19 vaccines in pediatric populations: Comirnaty (BNT162b2, also known as Pfizer BioNTech COVID-19 Vaccine) and Spikevax (mRNA-1273, also known as COVID-19 Moderna), for children aged 5–11 years [5], and Comirnaty exclusively for those aged 12–17 years [6]. Two distinct nationwide vaccination initiatives were launched in response to this decision, with the first, targeting adolescents aged 12–17 years, commencing in late October 2021 [7], and the second, focusing on children aged 5–11 years, which began in April 2022 [8,9].

The national objectives aimed to achieve complete two-dose vaccination coverage for all children aged 12–17 years by the conclusion of the first quarter in 2022, and to extend vaccination to all children aged 5–11 years by the end of August 2022 [10]. However, the first goal was not achieved until August 2022, five months later than the original plan [11]. By the end of 2022, the second target still remained unmet, with COVID-19 vaccine dose 1 and dose 2 coverage percentages for children aged 5–11 years at 92.4% and 73.8%, respectively [12]. For adults, in addition to Comirnaty and Spikevax, which were approved for pediatric use, seven other vaccines were also accepted for administration, including AstraZeneca (AZD1222), Covilo (SinoPharm COVID-19 Vaccine (Vero Cell), Inactivated), Abdala (CIGB-66), Covaxin (BBV512), Hayat-Vax (CNBG42, or also known as SARS-CoV-2 Vaccine (Vero Cell), Inactivated), Janssen (Ad26.COV2.S), and Sputnik-V (or Gam-COVID-Vac) [13].

To date, there have been no studies conducted in Vietnam to identify parental decision-making for their children to receive a COVID-19 vaccine and related factors after the vaccination campaigns rolled out for children. This study aims to understand the factors influencing parents’/guardians’ decision-making to vaccinate their children aged 6–17 years against COVID-19 in Thai Nguyen province, Vietnam, over the period of 2021–2023.

## 2. Materials and Methods

### 2.1. Study Design and Study Location

This is a cross-sectional study implemented through an online survey. The selected study site, Thai Nguyen province, is recognized as the political and economic center of the northern midland and mountainous region in Vietnam [14]. It is home to 51 ethnic groups, with the Kinh ethnic group accounting for the highest share, of about 70%, while various ethnic minorities make up the remaining 30%, contributing to cultural diversity [15,16]. The economies in this province are industry and construction (58%), followed by services (28%) and agriculture, forestry, and fishery (10%) [17]. The average Gross Regional Domestic Product (GRDP) per capita in Thai Nguyen in 2022 is VNM 110 million per year (equivalent to USD 4652), ranking first in the northern midland and mountainous region and tenth nationwide [18].

### 2.2. Study Participants and Sampling

**Figure 1:**
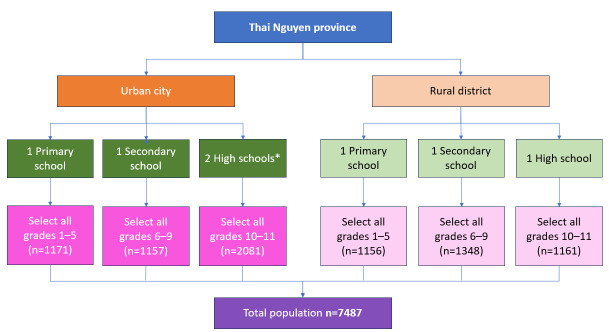
Flow chart of source population and sampling process. * In the initial design, the inclusion was limited to one high school located in the urban area. However, during the data collection phase, an additional high school located in urban city expressed voluntary interest in participating in our research and was included.

Figure 1 illustrates the methodology employed to select the study population for this study. The Thai Nguyen Center for Disease Control (CDC) purposefully selected one urban area, Thai Nguyen city, and one rural area, Phu Binh district, both situated in Thai Nguyen province. Within each of these areas, the Department of Education and Training purposefully selected one primary school, one secondary school, and one high school for our study. During the recruitment phase, an additional high school in Thai Nguyen city expressed interest in participating in our study to the Thai Nguyen CDC and was included, leading to a total of seven schools being included. In each selected school, we administered our survey to all parents or guardians of children aged 6 to 17 years, hereinafter referred to as ‘parents’. The total target population consisted of 7487 individuals. This was an exploratory study without formal hypothesis testing. Hence, no formal sample size calculations were performed.

The inclusion criteria were as follows: (1) participants had to be at least 18 years old, and (2) they were willing to take part in the study and provided informed consent. Individuals who were unable to complete or respond to the questionnaire were excluded from participation.

### 2.3. Data Collection

An online survey using REDCap was conducted between 13 April and 18 May 2023. In Vietnam, each school class has a group chat using a mobile phone application called Zalo, through which teachers send notifications to parents/guardians on several topics (e.g., exams, child health, COVID-19 alerts, etc.). We used this platform, with the full support of the Department of Education and Training and school principals, to circulate an anonymous online questionnaire to parents. Teachers sent an electronic participant information sheet and the survey link via the Zalo app to parents of all children in their respective classes. Two additional reminders were sent to parents at the end of the second and fourth weeks of the five-week data collection period.

### 2.4. Survey Structure

The survey was structured into six distinct sections and piloted. The first section collected demographic data from the parents, such as age, their relationship with the child (father/mother/other), residential setting (urban/rural), ethnicity (Kinh/other), highest level of education attained, primary occupation, marital status, and household size. The second section collected child-specific data, including the child’s age, gender, school type (primary/secondary/high), school location (urban/rural), the presence of underlying health conditions or chronic diseases (yes/no), and any history of COVID-19 infection (yes/no). The third section focused on elucidating decision-making employed by parents regarding the COVID-19 vaccination of their children, along with the primary reasons that informed their decision-making.

Following this, the fourth section further explored factors associated with parental decision-making and encompassed a set of twenty items that were conceptualized based on the Health Belief Model (HBM). The HBM was developed in the 1950s and posits that health-related behaviour is influenced by multiple factors, including ‘perceived severity’, ‘perceived susceptibility’, ‘perceived barriers’, ‘cues to action’, ‘perceived benefits’, and ‘self-efficacy’ [19]. The HBM stands as one of the most commonly employed models for comprehending COVID-19 vaccination behaviors [20]. In our study, participants ranked their agreement with twenty statements categorized under four groups, namely, ‘Perceived Susceptibility and Severity of COVID-19′, ‘Perceived Barriers’, ‘Perceived Benefits of COVID-19 Vaccine’, and ‘Cues to Action’, on a five-point Likert scale from strongly disagree to strongly agree.

The next section sought to ascertain parents’ personal experiences with COVID-19 infection and their vaccination history. The last section sought to encompass supplementary factors that could exert influence on decisions, including the COVID-19 experiences of other members within the family and the community, as well as the COVID-19 vaccination status of the siblings of the child, where applicable.

### 2.5. Statistical Analysis

After being extracted from REDCap, data were imported to STATA 17.0 (Stata-Corp, https://www.stata.com/ accessed on 19 May 2023) for cleaning and analysis. Descriptive analysis was carried out, with categorical variables being presented as frequencies and percentages, while continuous variables including age, number of household members, and number of siblings aged 5–17 years were summarized using medians and interquartile ranges. The Kruskal–Wallis test was used to compare medians between groups, while the chi-squared test and Fisher’s exact test were employed to assess differences in proportions where applicable. We constructed univariate and multivariable multinomial regression models to identify factors associated with parental decision-making, with socio-demographic characteristic variables with *p*-values < 0.05 in the univariate analysis being included in the multivariable models.

## 3. Results

A total of 4321 parents completed our survey, resulting in a response rate of 57.7%. Subsequently, during the data cleaning process, we identified and removed 86 records in which respondents self-identified as being under 18 years old. In the end, we included 4235 records in our analysis, with 2136 from the urban city and 2099 from the rural district (Table 1). The study participants had a median age of 41 years, with most (74.7%) aged 31–45. Mothers made up the majority (80.4%), and the participants were evenly split between urban (52.2%) and rural (47.8%) areas. Most were Kinh (88.0%), and education levels were evenly distributed between high school or lower (42.4%) and university or higher (42.2%). The largest occupational group was office staff or business (46.2%), followed by farmers (18.9%) and blue-collar workers (19.2%). Most of the participants were married or cohabitating (93.4%), with a median household size of four members (Table 2).

The multivariable analysis in Table 3 shows that younger parents (18–30 years) are significantly more likely to reject all doses of the COVID-19 vaccine for their children (aCoef. = −1.54, *p* < 0.01) compared to accepting some doses (aCoef. = −0.63, *p* < 0.01), indicating stronger hesitancy in this group. Similarly, urban parents are more likely to reject all doses (aCoef. = −1.04, *p* < 0.01) than to accept only some doses (aCoef. = −0.56, *p* < 0.01), suggesting greater vaccine reluctance in urban areas. Parents with higher education levels also show more likelihood of rejecting all doses (aCoef. = 0.43, *p* < 0.01) than of accepting some doses (aCoef. = 0.17, *p* = 0.02), indicating selective vaccination behaviors. In terms of occupation, farmers had the highest vaccine acceptance, with other occupational groups being more likely to reject all doses (aCoef. = 0.30, *p* = 0.01) or accept some doses (aCoef. = 0.21, *p* < 0.01), further emphasizing that farmers are more likely to fully vaccinate their children compared to other groups.

Table 4 presents the COVID-19- and vaccination-related characteristics of the parents and their families, while Table 5 provides the results of the multivariable analysis on how these characteristics influence parents’ decisions to vaccinate their children against COVID-19. Parents who were unvaccinated or had received only one dose were significantly more likely to reject all doses of the vaccine for their children (aCoef. = −2.25, *p* < 0.01) and to accept only some doses (aCoef. = −1.53, *p* < 0.01) compared to fully vaccinated parents. Additionally, parents with a history of COVID-19 were more likely to accept only some doses for their children (aCoef. = 0.29, *p* = 0.03), suggesting that prior infection influences partial vaccine acceptance. When it comes to information sources, the parents who received vaccination information from healthcare workers (aCoef. = −0.62, *p* < 0.01), relatives and friends (aCoef. = −0.41, *p* = 0.01), and official websites of the Ministry of Health and other health facilities (aCoef. = −0.43, *p* < 0.01) were significantly more likely to accept all doses of COVID-19 vaccine for their children.

Using the demographic and COVID-19-related characteristics of the children and their siblings presented in Table 6, we conducted a multivariable analysis (Table 7) to examine the impact of these factors on parents’ decisions to vaccinate their children against COVID-19. The results show that the parents with younger children (aged 6–11) were significantly more likely to reject all doses of the COVID-19 vaccine for their children compared to those with children aged 12–17 (aCoef. = −2.14, *p* < 0.01). The parents of children attending primary school were more likely to reject the vaccine—either partially or completely—compared to those whose children attended secondary or high school. Children with underlying conditions or chronic diseases were also associated with a higher likelihood of parental vaccine rejection (aCoef. = −0.60, *p* < 0.01). In contrast, parents with more than one child aged 5–17 were less likely to reject all doses (aCoef. = −0.36, *p* = 0.01), indicating that having additional children might positively influence acceptance. However, if the child’s siblings aged 5–17 were not fully vaccinated with the recommended doses, parents were more likely to reject COVID-19 vaccines for their child (aCoef. = −2.25, *p* < 0.01).

Table 8 and Table 9 provides the results of the univariable and multivariable analyses on four key domains of the HBM that influence parental decisions to vaccinate their children against COVID-19: perceived susceptibility and severity of COVID-19, perceived benefits of the vaccine, barriers related to knowledge and the community, and social cues.

- Perceived Susceptibility and Severity: The parents who believed their child was at high risk of contracting COVID-19 or thought their child could become severely ill from COVID-19 were significantly more likely to accept all COVID-19 vaccine doses for their children.
- Perceived Benefits: The parents who thought the vaccine would prevent their child from contracting COVID-19 or felt the vaccine helped protect others and relieved their own worries about illness were more likely to fully vaccinate their children.
- Perceived Barriers: The parents worried about potential side effects, believed that previous infection provided immunity, or thought COVID-19 could resolve without vaccination were more likely to reject all or some vaccine doses for their children. Additional barriers included fears of allergic reactions, concerns about chronic diseases worsening after vaccination, distrust in vaccination settings like schools, and negative stories from the community, all of which discouraged full vaccination.
- Cues to Action: The parents who believed in the importance of herd immunity were significantly more likely to vaccinate their children fully. Recommendations from healthcare workers and encouragement from teachers also positively impacted parental vaccination decisions. Additionally, seeing other parents vaccinate their children or knowing that unvaccinated children might face barriers to learning further motivated parents to fully vaccinate their children.

## 4. Discussion

Our study revealed a high overall acceptance rate among parents in Thai Nguyen province, Vietnam, for having their children vaccinated against COVID-19. We also identified several influential factors that were associated with these vaccination decisions. There have been numerous studies on parental hesitancy towards COVID-19 vaccination for their children conducted in diverse countries worldwide. We focus on discussing our results with those of studies implemented in low- and middle-income countries because of the similarities between the settings.

In our study, we observed that 81.3% of parents opted for their children to receive all COVID-19 vaccine doses, aligning closely with the 82.6% acceptance rate reported in a study conducted across 31 provinces in the Chinese mainland [21]. This acceptance rate surpasses those recorded in Jordan, where rates ranged from 25.4% [22] to 30.2% [23], as well as in Ghana, where the acceptance rate stood at 73.3% [24]. The higher acceptance rate in Vietnam could be attributed to the country’s strong public health infrastructure and a centralized vaccination campaign, which promoted trust in government-led health initiatives. Additionally, Vietnam’s cultural emphasis on collective responsibility regarding hygiene and public health, and the effectiveness of local healthcare systems in delivering vaccines, may have contributed to higher acceptance rates. In contrast, Jordan and Ghana may face more significant barriers, such as weaker healthcare systems, lower trust in government health policies, and logistical challenges in rural areas. Notably, two separate studies in China reported higher acceptance rates among parents, of 88.7% [25] and 92.2% [26]. In our study, the proportions of parents who consented to administering all COVID-19 vaccine doses to their children aged 6–11 years and 12–17 years were 66.5% and 86.7%, respectively. These rates slightly exceeded the figures observed among parents of children in the age groups of 0–11 years and 12–17 years in a study implemented in Thailand, where the corresponding percentages were 43.1% and 82.9% [27]. The disparities observed between the outcomes of our investigation and those of previous studies may be attributed to variations in the age groups of the children under consideration in each study, the utilization of different vaccine formulations within each country, and the distinct contextual factors prevalent in each study setting.

In Vietnam, two previous studies on parental vaccine acceptance were conducted prior to the commencement of COVID-19 vaccination campaigns for children [28,29]. The initial study, carried out between 21 January and 20 April 2021, involved 1015 parents with children aged 5–17 years, who sought care at four community health centers and hospitals across two districts in Ho Chi Minh City, Vietnam. This study reported an acceptance rate of 73.8%, lower than our study’s finding of 81.3% [28]. The second investigation, encompassing 5357 parents of children aged 5–11 years throughout Vietnam, was conducted between 20 February and 6 March 2022, preceding the official launch of the COVID-19 vaccination campaign for this age group in Vietnam, in April 2022. That study revealed that only 36% of parents were inclined to have their children vaccinated [29], whereas we found the acceptance rate among parents with children in this age group to be significantly higher, at 66.5%. These disparities between our research and other studies might be explained by differences in study contexts (i.e., whether the studies were conducted before or after the launch of vaccination campaigns), variations in sample sizes, and variations in study locations. Another contributing factor may be the effective communication strategies and public health policies implemented in Vietnam, which have significantly enhanced parental acceptance of vaccination.

Our study identified several significant factors influencing parental acceptance of COVID-19 vaccination for their children, including parental characteristics, experiences, and the attributes of the children involved. Regarding parental age, we found that younger parents, aged 18–30, were less likely to accept all vaccine doses for their children (64.2%) compared to their older counterparts (79.5–88.5%). This finding contrasts with previous research conducted in Jordan, where parents younger than 30 years were more willing to vaccinate their children against COVID-19 (32.5%) compared to those aged 30–40 years and older than 40 years (21.2% and 28.8%, respectively) [22]. The differences in findings between the two studies may be attributed to cultural variations, differing levels of vaccine awareness and trust in healthcare systems, and the impact of local public health campaigns. In particular, the hesitancy observed among younger parents in our study could have been influenced by their reliance on social media as a primary source of information. Research indicates that younger individuals are more likely to encounter misinformation online, which may shape their perceptions of vaccine safety and efficacy [30,31]. Therefore, targeted public health communication campaigns specifically for younger parents are needed in Vietnam to address their concerns and improve vaccine confidence, focusing on the impact of social media and misinformation.

In terms of parental education and living area, our study found that parents with higher education levels and those living in the urban city exhibited lower acceptance of COVID-19 vaccines for their children. A similar pattern was observed in a study from Thailand, where parents with higher education levels showed more vaccine hesitancy compared to those with lower education levels [32]. This trend may be due to higher-educated individuals being more critical of vaccine information and more likely to encounter diverse sources of information, including misinformation. To enhance COVID-19 vaccine acceptance among parents, especially those with higher education in urban areas, it is essential to implement targeted educational campaigns, engage with misinformation through credible resources, collaborate with trusted community leaders, leverage social media for positive messaging, establish monitoring mechanisms for parental attitudes, and share personal testimonials from vaccinated parents.

Our analysis revealed that parents who had received multiple COVID-19 vaccine doses exhibited higher acceptance rates for vaccinating their children compared to those who were unvaccinated or who had received only one dose. This finding aligns with studies conducted in China and Thailand, which also observed that parents hesitant over vaccinating themselves were similarly hesitant over vaccinating their children [21,26,33]. One possible explanation is that parents who are fully vaccinated themselves have greater trust in the vaccine’s safety and efficacy, which positively influences their decision to vaccinate their children. Conversely, unvaccinated parents may harbor doubts or fears about the vaccine, leading to reluctance over vaccinating their children. To address this, public health initiatives should focus on increasing vaccination rates among parents, as well as implementing targeted educational campaigns that highlight the importance of vaccination for both parents and their children.

Our study found that parents were more likely to vaccinate older children (12–17 years) than younger children (6–11 years). This finding aligns with a study from Thailand, which reported higher COVID-19 vaccine hesitancy among parents of children under 12 compared to those with older children [27]. One possible explanation for this is that parents may believe that younger children’s bodies are less capable of handling new vaccines compared to those of older children. Parents might have concerns about younger children’s immune systems and their ability to cope with potential side effects, leading to greater hesitancy over vaccinating younger children. To mitigate this hesitancy, public health campaigns should focus on providing clear, evidence-based information about the safety and efficacy of COVID-19 vaccines for younger children, as well as addressing parental concerns directly through community engagement and education.

The findings of our study indicate that parents whose children have underlying conditions, chronic diseases, or allergies are more likely to reject COVID-19 vaccinations for their children. This is consistent with the findings from a study in China, which found that hesitant parents were more likely to be those with children having allergic issues [25]. To address this hesitancy, public health initiatives should prioritize delivering targeted, evidence-based information that addresses the specific concerns of these parents, particularly regarding the safety and efficacy of vaccinations for children with underlying health issues. Engaging healthcare professionals to facilitate discussions and provide reassurance could also help improve vaccine acceptance in this vulnerable group.

Additionally, our investigation highlights the significant role of health beliefs in influencing parental decisions. Parents who perceived their children as being at high risk for COVID-19 or who believed in the severity of potential illness and the benefits of vaccination were more likely to accept all doses for their children. In contrast, concerns regarding various barriers, such as fears of side effects and misconceptions about natural immunity, contributed to increased vaccine hesitancy among the parents. Similar findings were also identified in other research in low- and middle-income settings [21,22,23,24,26,27,32]. To enhance vaccine acceptance, it is essential to improve communication strategies that raise parental awareness across all mentioned domains. Engaging healthcare workers, teachers, and credible sources of information, such as the websites of the Ministry of Health and other reputable health facilities, can serve as effective channels for disseminating accurate information and fostering trust in vaccination programs, as our study indicated.

Several limitations should be acknowledged in this study. Firstly, convenience sampling to select the schools for our study may have introduced selection bias, which may have affected both covariates and outcome estimates in our sample compared to a representative population sample. Secondly, the response rate in our study was just under 60%, which might have contributed to selection bias. In our study, over 80% of the participants were female, a rate that was significantly higher than the 2023 female share of the general population in this province, which stood at 51.2% [34]. This discrepancy may be attributed to cultural norms in Vietnam, where mothers typically take an active role in their children’s education and health-related decisions, leading to greater involvement in vaccination discussions. Thirdly, this study focused on a single province in Northern Vietnam, limiting the generalizability of our findings to other regions. This limitation is significant given the diversity of contexts across different provinces. Fourthly, online data collection may also introduce selection bias, as parents in rural areas with limited access to digital devices or stable internet connections might have been unable to receive or complete the survey. Notwithstanding the limitations, our study contributes a novel finding regarding the proportion of parents who accepted some but not all COVID-19 vaccine doses for their children, a facet not addressed in the existing literature. Furthermore, we examined various factors associated with parental decisions, including parental characteristics, their experiences with COVID-19 infection and vaccination, child-related attributes, and social influences such as information sources and cues for action. Understanding these diverse elements can provide valuable insights into the dynamics of vaccine acceptance among parents.

## 5. Conclusions

Our study reveals a high overall acceptance rate among parents for having their children vaccinated against COVID-19 in Thai Nguyen province, Vietnam. Several factors were identified as potential determinants influencing parental decision-making processes, including parental age, education, occupation, area of residence, health beliefs, prior experiences with COVID-19 infection and vaccination, and the ages and underlying conditions of the children. To enhance COVID-19 vaccination acceptance, targeted communication strategies should focus on younger parents, those living in urban areas, parents with higher educational levels, and those with children who are younger or have underlying medical conditions. Trusted sources such as healthcare workers, teachers, and official health websites are essential for disseminating accurate information and fostering trust in vaccination programs.

## Figures and Tables

**Table 1:** Distribution of study participants in relation to source population.

Characteristics of Parents with Children in Urban and Rural Schools	Urban City		Rural District	
	N	Study Participant (*n*, %)	N	Study Participant (*n*, %)
Primary school	1171	486 (41.5%)	1156	638 (55.2%)
Secondary school	1157	328 (28.3%)	1348	628 (46.6%)
High school	2081	1322 (63.5%)	1161	833 (71.7%)
Total	4409	2136 (48.4%)	3665	2099 (57.3%)

**Table 2:** Socio-demographic characteristics of parents.

Characteristics	Total	Accepted All Doses	Accepted Some but Not All Doses	Rejected All Doses	Did Not Vaccinate for Other Reasons	*p*-Value
	*n* (%)	*n* (%)	*n* (%)	*n* (%)	*n* (%)	
Total	4235 (100)	3444 (81.3)	409 (9.7)	193 (4.6)	189 (4.5)	
Age (Median, IQR)	41 (38–45)	42 (38–46)	40 (36–43)	37 (27–40)	40 (36–44)	<0.01 *
Age group						
18–30	95 (2.3)	61 (64.2)	13 (13.7)	10 (10.5)	11 (11.6)	<0.01 ^#^
31–45	3125 (74.7)	2485 (79.5)	325 (10.4)	171 (5.5)	144 (4.6)	
46+	962 (23.0)	851 (88.5)	67 (7.0)	11 (1.1)	3 (3.4)	
Relationship with the child						<0.01 ^†^
Father	788 (18.6)	685 (86.9)	61 (7.7)	25 (3.2)	17 (2.2)	
Mother	3403 (80.4)	2723 (80.0)	343 (10.1)	167 (4.9)	170 (5.0)	
Other	44 (1.0)	36 (81.8)	5 (11.4)	1 (2.3)	2 (4.6)	
Area of residence						<0.01 ^#^
Urban	2210 (52.2)	1654 (74.8)	272 (12.3)	153 (6.9)	131 (5.9)	
Rural	2025 (47.8)	1790 (88.4)	137 (6.8)	40 (2.0)	58 (2.9)	
Ethnicity						0.051 ^#^
Kinh	3696 (88.0)	3014 (81.6)	366 (9.9)	161 (4.4)	155 (4.2)	
Other	502 (12.0)	399 (79.5)	42 (8.4)	30 (6.0)	31 (6.2)	
Highest education						<0.01 ^#^
High school or lower	1766 (42.4)	1567 (88.7)	119 (6.7)	35 (2.0)	45 (2.6)	
Vocational school	645 (15.5)	510 (79.1)	71 (11.0)	27 (4.2)	37 (5.7)	
University or higher	1757 (42.2)	1311 (74.6)	214 (12.2)	131 (7.5)	101 (5.8)	
Occupation						<0.01 ^#^
Farmer	794 (18.9)	730 (91.9)	42 (5.3)	6 (0.8)	16 (2.0)	
Blue-collar worker	804 (19.2)	705 (87.7)	54 (6.7)	19 (2.4)	26 (3.2)	
Office staff/ Business	1939 (46.2)	1456 (75.1)	235 (12.1)	138 (7.1)	110 (5.7)	
Other	661 (15.8)	521 (78.8)	77 (11.7)	30 (4.5)	33 (5.0)	
Marital status						0.31 ^#^
Married/Cohabitation	3910 (93.4)	3174 (81.2)	380 (9.7)	177 (4.5)	179 (4.6)	
Divorced/Separated/Widowed/Single	275 (6.6)	230 (83.6)	23 (8.4)	15 (5.5)	7 (2.6)	
Number of household members (Median, IQR)	4 (4–5)	4 (4–5)	4 (4–5)	4 (4–5)	4 (4–5)	0.43 *

* *p*-value from Kruskal–Wallis test; ^#^ *p*-value from chi-squared test; ^†^ *p*-value from Fisher’s exact test.

**Table 3:** Multivariable analysis of socio-demographic characteristics of parents.

Predictor	Accepted Some Doses vs. Accepted All Doses			Rejected All Doses vs. Accepted All Doses			Did Not Vaccinate for Other Reasons vs. Accepted All Doses		
	aCoef.	SE	*p*-Value	aCoef.	SE	*p*-Value	aCoef.	SE	*p*-Value
Parent’s age group	−0.63	0.13	<0.01	−1.54	0.21	<0.01	−0.70	0.19	<0.01
Relationship with the child	0.04	0.15	0.77	−0.22	0.21	0.29	0.50	0.24	0.04
Area of residence	−0.56	0.13	<0.01	−1.04	0.21	<0.01	−0.70	0.19	<0.01
Ethnicity	−0.32	0.18	0.07	0.08	0.21	0.71	0.20	0.21	0.34
Highest education	0.17	0.07	0.02	0.43	0.11	<0.01	0.19	0.10	0.053
Occupation	0.21	0.07	<0.01	0.30	0.12	0.01	0.20	0.11	0.06
Marital status	−0.23	0.24	0.35	0.03	0.31	0.91	−0.92	0.46	0.048

aCoef.: Multinomial regression coefficient adjusted for parent’s age group, relationship with the child, area of residence, highest education, and occupation. SE: Standard error.

**Table 4:** COVID-19 and vaccination-related characteristics of parents and families.

Characteristics	Total	Accepted All Doses	Accepted Some but Not All Doses	Rejected all Doses	Did Not Vaccinate for Other Reasons	*p*-Value
	*n* (%)	*n* (%)	*n* (%)	*n* (%)	*n* (%)	
COVID-19 vaccination status of parents						<0.01 ^†^
Unvaccinated * or received 1 dose	63 (1.5)	33 (52.4)	14 (22.2)	12 (19.1)	4 (6.4)	
Received 2 doses or more	4172 (98.5)	3411 (81.8)	395 (9.5)	181 (4.3)	185 (4.4)	
Parent received mixed types of COVID-19 vaccines						0.53 ^#^
Yes	1335 (37.9)	1054 (79.0)	143 (10.7)	71 (5.3)	67 (5.0)	
No	2188 (62.1)	1770 (80.9)	217 (9.9)	99 (4.5)	102 (4.7)	
Parents received only one type of COVID-19 vaccine						
Only AstraZeneca						0.02 ^#^
Yes	640 (15.1)	502 (78.4)	59 (9.2)	38 (5.9)	41 (6.4)	
No	3595 (84.9)	2942 (81.8)	350 (9.7)	155 (4.3)	148 (4.1)	
Only Comirnaty (Pfizer)						0.11 ^#^
Yes	1249 (29.5)	1031 (82.6)	126 (10.1)	45 (3.6)	47 (3.8)	
No	2986 (70.5)	2413 (80.8)	283 (9.5)	148 (5.0)	142 (4.8)	
Only Moderna						0.95 ^#^
Yes	200 (4.7)	160 (80.0)	21 (10.5)	9 (4.5)	10 (5.0)	
No	4035 (95.3)	3284 (81.4)	388 (9.6)	184 (4.6)	179 (4.4)	
Only Vero Cell						0.38 ^#^
Yes	91 (2.2)	70 (76.9)	11 (12.1)	7 (7.7)	3 (3.3)	
No	4144 (97.9)	3374 (81.4)	398 (9.6)	186 (4.5)	186 (4.5)	
Hospitalized within 7 days of receiving COVID-19 vaccine						0.82 ^#^
Yes	392 (9.3)	313 (79.9)	43 (11.0)	18 (4.6)	18 (4.6)	
No	3828 (4220)	3124 (81.6)	366 (9.6)	168 (4.4)	170 (4.4)	
COVID-19 history of parent						<0.01 ^#^
Yes	3044 (71.9)	2406 (79.0)	325 (10.7)	158 (5.2)	155 (5.1)	
No	1191 (28.1)	1038 (87.2)	84 (7.1)	35 (2.9)	34 (2.9)	
COVID-19 severity of parent						0.03 ^†^
No symptoms	233 (7.7)	201 (86.3)	15 (6.4)	11 (4.7)	6 (2.6)	
Had symptoms, self-treatment at home	2652 (87.1)	2070 (78.1)	295 (11.1)	143 (5.4)	144 (5.4)	
Had symptoms and visited health facilities for treatment	159 (5.2)	135 (84.9)	15 (9.4)	4 (2.5)	5 (3.1)	
Family members hospitalized because of COVID-19						0.68 ^#^
Yes	166 (4.0)	135 (81.3)	16 (9.6)	10 (6.0)	5 (3.0)	
No	3985 (96.0)	3244 (81.4)	386 (9.7)	180 (4.5)	175 (4.4)	
Area of residence under quarantine to prevent COVID-19 infection						0.06 ^#^
Yes	1048 (25.2)	873 (83.3)	102 (9.7)	34 (3.2)	39 (3.7)	
No	3106 (74.8)	2505 (80.7)	301 (9.7)	156 (5.0)	144 (4.6)	
COVID-19 vaccination information sources						
Healthcare workers						<0.01 ^#^
Yes	2453 (57.9)	2048 (83.5)	216 (8.8)	84 (3.4)	105 (4.3)	
No	1782 (42.1)	1396 (78.3)	193 (10.8)	109 (6.1)	84 (4.7)	
TV/ Radio						0.01 ^#^
Yes	2856 (67.4)	2281 (79.9)	299 (10.5)	139 (4.5)	137 (4.8)	
No	1379 (32.6)	1163 (84.3)	110 (8.0)	54 (3.9)	52 (3.8)	
Social media						<0.01 ^#^
Yes	2375 (56.1)	1879 (79.1)	248 (10.4)	126 (5.3)	122 (5.1)	
No	1860 (43.9)	1565 (84.1)	161 (8.7)	67 (3.6)	67 (3.6)	
Relatives and friends						0.02 ^#^
Yes	1843 (43.5)	1501 (81.4)	163 (8.8)	79 (4.3)	100 (5.4)	
No	2392 (56.5)	1943 (81.2)	246 (10.3)	114 (4.8)	89 (3.7)	
Website of MOH and other health facilities						0.01 ^#^
Yes	2273 (53.7)	1856 (81.7)	193 (8.5)	110 (4.8)	114 (5.0)	
No	1962 (46.3)	1588 (80.9)	216 (11.0)	83 (4.2)	75 (3.8)	
Other sources						0.13 ^†^
Yes	87 (2.1)	77 (88.5)	3 (3.5)	5 (5.8)	2 (2.3)	
No	4148 (98.0)	3367 (81.2)	406 (9.8)	188 (4.5)	187 (4.5)	

^#^ *p*-value from chi-squared test; ^†^ *p*-value from Fisher’s exact test; * Unvaccinated: Received 0 doses of any vaccines.

**Table 5:** Multivariable analysis of COVID-19 and vaccination-related characteristics of parents and families.

Predictor	Accepted Some Doses vs. Accepted All Doses			Rejected All Doses vs. Accepted All Doses			Did Not Vaccinate for Other Reasons vs. Accepted All Doses		
	aCoef.	SE	*p*-Value	aCoef.	SE	*p*-Value	aCoef.	SE	*p*-Value
COVID-19 vaccination status of parents	−1.53	0.34	<0.01	−2.25	0.38	<0.01	−1.07	0.55	0.050
Parent receiving mixed types of COVID-19 vaccines	0.06	0.08	0.48	−0.05	0.12	0.68	0.07	0.12	0.58
Parent receiving only AstraZeneca vaccine	0.06	0.11	0.60	0.30	0.16	0.051	0.20	0.16	0.21
Parent receiving only Comirnaty (Pfizer) vaccine	0.08	0.11	0.50	−0.44	0.15	<0.01	−0.28	0.16	0.07
Parent receiving only Moderna vaccine	−0.13	0.14	0.35	−0.23	0.20	0.24	0.17	0.19	0.35
Parent receiving only Vero Cell vaccine	0.11	0.19	0.55	0.45	0.25	0.07	0.26	0.26	0.33
Parents being hospitalized within 7 days of receiving COVID-19 vaccine	−0.12	0.18	0.48	0.02	0.26	0.93	0.09	0.27	0.74
COVID-19 history of parent	0.29	0.13	0.03	0.28	0.20	0.16	0.37	0.20	0.06
COVID-19 severity of parent	0.34	0.18	0.06	−0.05	0.26	0.84	0.19	0.26	0.47
Family members hospitalized because of COVID-19	0.07	0.28	0.79	−0.16	0.35	0.64	0.39	0.47	0.40
Area of residence under quarantine to prevent COVID-19 infection	0.01	0.12	0.93	0.49	0.20	0.02	0.17	0.19	0.37
COVID-19 vaccination information sources									
Healthcare workers	−0.24	0.11	0.02	−0.62	0.15	<0.01	−0.10	0.16	0.52
TV/Radio	0.10	0.13	0.45	−0.20	0.18	0.26	−0.02	0.18	0.92
Social media	0.09	0.11	0.41	0.09	0.16	0.58	0.21	0.16	0.20
Relatives and friends	−0.32	0.11	<0.01	−0.41	0.16	0.01	0.19	0.16	0.23
Websites of MOH and other health facilities	−0.43	0.11	<0.01	−0.17	0.16	0.27	0.08	0.16	0.61
Other source	−1.18	0.59	0.047	0.10	0.48	0.84	−0.76	0.72	0.29

aCoef.: Multinomial regression coefficient adjusted for parent’s age group, relationship with the child, area of residence, highest education, and occupation. SE: Standard error.

**Table 6:** Characteristics of children and their siblings.

Characteristics	Total	Accepted All Doses	Accepted Some but Not All Doses	Rejected All Doses	Did Not Vaccinate for Other Reasons	*p*-Value
	*n* (%)	*n* (%)	*n* (%)	*n* (%)	*n* (%)	
Child’s age (median, IQR)	16 (11–17)	16 (12–17)	13 (10–16)	9 (8–12)	12 (9–16)	<0.01 *
Child’s age group						<0.01 ^#^
6–11	1118 (26.4)	743 (66.5)	155 (13.9)	137 (12.3)	83 (7.4)	
12–17	3117 (73.6)	2701 (86.7)	254 (8.2)	56 (1.8)	106 (3.4)	
Child’s sex						0.62 ^#^
Male	2022 (74.7)	1634 (80.8)	196 (9.7)	101 (5.0)	91 (4.5)	
Female	2213 (52.3)	1810 (81.8)	213 (9.6)	92 (4.2)	98 (4.4)	
School type						<0.01 ^#^
Primary	1124 (26.5)	746 (66.4)	156 (13.9)	137 (12.2)	85 (7.6)	
Secondary	956 (22.6)	780 (81.6)	96 (10.0)	37 (3.9)	43 (4.5)	
High	2155 (50.9)	1918 (89.0)	157 (7.3)	19 (0.9)	61 (2.8)	
School location						<0.01 ^#^
Urban	2136 (50.4)	1594 (74.6)	262 (12.3)	150 (7.0)	130 (6.1)	
Rural	2099 (49.6)	1850 (88.1)	147 (7.0)	43 (2.1)	59 (2.8)	
The child has underlying conditions/chronic diseases						<0.01 ^#^
Yes	557 (13.2)	416 (74.7)	57 (10.2)	40 (7.2)	44 (7.9)	
No	3678 (86.9)	3028 (82.3)	352 (9.6)	153 (4.2)	145 (3.9)	
COVID-19 history of the child						<0.01 ^†^
Yes	2745 (62.0)	2180 (79.4)	283 (10.3)	138 (5.0)	144 (5.3)	
No	1490 (35.2)	1264 (84.8)	126 (8.5)	55 (3.7)	45 (3.0)	
Number of siblings aged 5–17						
None	1208 (30.9)	967 (80.1)	117 (9.7)	72 (6.0)	52 (4.3)	0.09 ^#^
One	2178 (55.7)	1760 (80.8)	215 (9.9)	96 (4.4)	107 (4.9	
Two or more	528 (13.5)	445 (84.3)	49 (9.3)	16 (3.0)	18 (3.4)	
COVID-19 vaccination of child’s sibling aged 5–17						<0.01 ^#^
All siblings were vaccinated all recommended doses by MOH	2000 (65.7)	1773 (88.9)	151 (7.6)	22 (1.1)	54 (2.7)	
All siblings were unvaccinated or not fully vaccinated	1043 (34.3)	713 (68.4)	145 (13.9)	101 (9.7)	84 (8.1)	

* *p*-value from Kruskal–Wallis test; ^#^ *p*-value from chi-squared test; ^†^ *p*-value from Fisher’s exact test.

**Table 7:** Multivariable analysis of characteristics of children and their siblings.

Predictor	Accepted Some Doses vs. Accepted All Doses			Rejected All Doses vs. Accepted All Doses			Did Not Vaccinate for Other Reasons vs. Accepted All Doses		
	aCoef.	SE	*p*-Value	aCoef.	SE	*p*-Value	aCoef.	SE	*p*-Value
Child’s age group	−0.76	0.12	<0.01	−2.14	0.18	<0.01	−1.05	0.17	<0.01
Child’s sex	−0.00	0.11	0.99	−0.14	0.15	0.36	−0.02	0.15	0.90
School type	−0.51	0.07	<0.01	−1.46	0.12	<0.01	−0.72	0.10	<0.01
School location	0.13	0.36	0.71	−0.23	0.59	0.70	−1.00	0.74	0.17
The child has underlying conditions/ chronic diseases	−0.15	0.16	0.34	−0.60	0.19	<0.01	−0.67	0.19	<0.01
COVID-19 history of the child	0.10	0.12	0.37	0.10	0.17	0.57	0.44	0.18	0.02
Number of siblings aged 5–17	−0.02	0.09	0.86	−0.36	0.13	0.01	0.00	0.13	0.03
COVID-19 vaccination of child’s sibling aged 5–17	−0.79	0.13	<0.01	−2.25	0.24	<0.01	−1.11	0.19	<0.01

aCoef.: Multinomial regression coefficient adjusted for parent’s age group, relationship with the child, area of residence, highest education, and occupation. SE: Standard error.

**Table 8:** Univariable analysis of Health Belief Model items.

	Predictor	Accepted Some Doses vs. Accepted All Doses			Rejected All Doses vs. Accepted All Doses			Did Not Vaccinate for Other Reasons vs. Accepted All Doses		
		Coef.	SE	*p*-Value	Coef.	SE	*p*-Value	Coef.	SE	*p*-Value
1	Perceived Susceptibility and Severity of COVID-19									
1.1	My child is at high risk for future COVID-19 infection/re-occurrence (n = 4020).	−0.14	0.06	0.01	−0.21	0.08	0.01	0.14	0.09	0.11
1.2	My child could be severely ill if he/she got COVID-19 (n = 4025).	−0.30	0.05	<0.01	−0.36	0.07	<0.01	−0.11	0.08	0.17
2	Perceived Benefits of COVID-19 vaccine									
2.1	Vaccination will help prevent my child from contracting COVID-19 (n = 4073).	−0.46	0.05	<0.01	−0.74	0.07	<0.01	−0.26	0.08	<0.01
2.2	By being immunized against COVID-19, my child will protect others from COVID-19 (n = 4152).	−0.30	0.07	<0.01	−0.71	0.08	<0.01	−0.09	0.11	0.43
2.3	When my child was vaccinated against COVID-19, I felt less worried about my child’s potential for serious illness (n = 4090).	−0.48	0.06	<0.01	−0.76	0.08	<0.01	−0.15	0.09	0.10
2.4	My child has been diagnosed with long COVID-19 symptoms, and I think COVID-19 vaccine can help relieve these symptoms (n = 3926).	−0.26	0.05	<0.01	−0.51	0.07	<0.01	−0.06	0.08	0.44
3	Perceived Barriers									
3.1	I am concerned that the COVID-19 vaccine may cause adverse events following the injection (n = 4060).	0.72	0.07	<0.01	0.90	0.11	<0.01	0.32	0.09	<0.01
3.2	COVID-19 infection can be self-limiting without vaccination (n = 3899).	0.61	0.06	<0.01	1.00	0.09	<0.01	0.32	0.08	<0.01
3.3	My child already has COVID-19 and has antibodies, so he/she does not need to be vaccinated anymore (n = 3945).	0.63	0.06	<0.01	0.83	0.08	<0.01	0.34	0.09	<0.01
3.4	My child is allergic to certain medications, and I am afraid that he/she can be allergic to COVID-19 vaccine too (n = 3990).	0.40	0.07	<0.01	0.57	0.10	<0.01	0.42	0.10	<0.01
3.5	My child has chronic diseases, I think COVID-19 vaccine can make him/her worse (n = 4002).	0.40	0.07	<0.01	0.40	0.10	<0.01	0.16	0.10	0.09
3.6	I do not feel safe because my child was told to get COVID-19 vaccinations at schools or some places that do not have enough infrastructure to manage emergency cases (n = 4054).	0.27	0.06	<0.01	0.36	0.08	<0.01	0.22	0.08	0.01
3.7	I heard terrible news/stories about getting the COVID-19 vaccine from my relatives and community (n = 3803).	0.30	0.06	<0.01	0.34	0.08	<0.01	0.03	0.08	0.70
4	Cues to Action									
4.1	I think that all people should be vaccinated against COVID-19 to increase the herd immunity (n = 4152).	−0.45	0.07	<0.01	−0.89	0.08	<0.01	−0.33	0.10	<0.01
4.2	I agree to have my child receiving COVID-19 vaccine because healthcare workers recommended it (n = 4083).	−0.30	0.07	<0.01	−0.50	0.09	<0.01	−0.24	0.10	0.02
4.3	Almost all people around me let their children get COVID-19 vaccine (n = 4111).	−0.67	0.06	<0.01	−1.20	0.08	<0.01	−0.62	0.09	<0.01
4.4	If my child does not get the COVID-19 vaccine, there will be some barriers to his or her learning (n = 3935).	−0.30	0.06	<0.01	−0.77	0.08	<0.01	−0.27	0.08	<0.01
4.5	Other vaccines have made my child less likely to get sick, so I believe the COVID-19 vaccine is effective in protecting him/her (n = 4035).	−0.28	0.06	<0.01	−0.65	0.08	<0.01	−0.11	0.09	0.24
4.6	I decided to have my child vaccinated because there are many children with COVID-19 at my child’s school or the surrounding area (n = 3973).	−0.11	0.06	0.06	−0.17	0.08	0.048	−0.10	0.08	0.22
4.7	The teachers in the school encourage and advise my child to get vaccinated against COVID-19 (n = 4114).	−0.19	0.07	0.01	−0.25	0.10	0.01	−0.00	0.11	0.99

Coef.: Multinomial regression coefficient. SE: Standard error.

**Table 9:** Multivariable analysis of Health Belief Model items.

	Predictor	Accepted Some Doses vs. Accepted All Doses			Rejected All Doses vs. Accepted All Doses			Not Vaccinating for Other Reasons vs. Accepted All Doses		
		aCoef.	SE	*p*-Value	aCoef.	SE	*p*-Value	aCoef.	SE	*p*-Value
1	Perceived Susceptibility and Severity of COVID-19									
1.1	My child is at high risk for future COVID-19 infection/re-occurrence (n = 4020).	−0.18	0.06	<0.01	−0.31	0.08	<0.01	0.08	0.09	0.38
1.2	My child could be severely ill if he/she got COVID-19 (n = 4025).	−0.30	0.06	<0.01	−0.39	0.08	<0.01	−0.12	0.08	0.14
2	Perceived Benefits of COVID-19 vaccine									
2.1	Vaccination will help prevent my child from contracting COVID-19 (n = 4073).	−0.42	0.06	<0.01	−0.71	0.08	<0.01	−0.21	0.08	0.01
2.2	By being immunized against COVID-19, my child will protect others from COVID-19 (n = 4152).	−0.35	0.07	<0.01	−0.87	0.09	<0.01	−0.12	0.12	0.30
2.3	When my child was vaccinated against COVID-19, I felt less worried about my child’s potential for serious illness (n = 4090).	−0.49	0.06	<0.01	−0.82	0.08	<0.01	−0.14	0.10	0.14
2.4	My child has been diagnosed with long COVID-19 symptoms, and I think COVID-19 vaccine can help relieve these symptoms (n = 3926).	−0.23	0.06	<0.01	−0.50	0.08	<0.01	−0.04	0.08	0.65
3	Perceived Barriers									
3.1	I am concerned that the COVID-19 vaccine may cause adverse events following the injection (n = 4060).	0.74	0.08	<0.01	0.93	0.12	<0.01	0.31	0.10	<0.01
3.2	COVID-19 infection can be self-limiting without vaccination (n = 3899).	0.57	0.06	<0.01	0.94	0.09	<0.01	0.26	0.09	<0.01
3.3	My child already has COVID-19 and has antibodies, so he/she does not need to be vaccinated anymore (n = 3945).	0.68	0.06	<0.01	0.91	0.09	<0.01	0.36	0.10	<0.01
3.4	My child is allergic to certain medications, and I am afraid that he/she can be allergic to COVID-19 vaccine too (n = 3990).	0.40	0.07	<0.01	0.57	0.11	<0.01	0.39	0.10	<0.01
3.5	My child has chronic diseases, I think COVID-19 vaccine can make him/her worse (n = 4002).	0.44	0.08	<0.01	0.47	0.11	<0.01	0.19	0.10	0.053
3.6	I do not feel safe because my child was told to get COVID-19 vaccinations at schools or some places that do not have enough infrastructure to manage emergency cases (n = 4054).	0.23	0.06	<0.01	0.27	0.09	<0.01	0.17	0.08	0.045
3.7	I heard terrible news/stories about getting the COVID-19 vaccine from my relatives and community (n = 3803).	0.33	0.06	<0.01	0.37	0.09	<0.01	0.05	0.09	0.55
4	Cues to Action									
4.1	I think that all people should be vaccinated against COVID-19 to increase the herd immunity (n = 4152).	−0.46	0.07	<0.01	−1.03	0.09	<0.01	−0.32	0.11	<0.01
4.2	I agree to have my child receiving COVID-19 vaccine because healthcare workers recommended it (n = 4083).	−0.32	0.07	<0.01	−0.57	0.10	<0.01	−0.27	0.11	0.01
4.3	Almost all people around me let their children get COVID-19 vaccine (n = 4111).	−0.63	0.07	<0.01	−1.23	0.09	<0.01	−0.57	0.09	<0.01
4.4	If my child does not get the COVID-19 vaccine, there will be some barriers to his or her learning (n = 3935).	−0.24	0.06	<0.01	−0.72	0.08	<0.01	−0.23	0.08	0.01
4.5	Other vaccines have made my child less likely to get sick, so I believe the COVID-19 vaccine is effective in protecting him/her (n = 4035).	−0.31	0.07	<0.01	−0.76	0.09	<0.01	−0.15	0.10	0.13
4.6	I decided to have my child vaccinated because there are many children with COVID-19 at my child’s school or the surrounding area (n = 3973).	−0.13	0.06	0.03	−0.24	0.09	0.01	−0.13	0.09	0.15
4.7	The teachers in the school encourage and advise my child to get vaccinated against COVID-19 (n = 4114).	−0.20	0.08	0.01	−0.33	0.11	<0.01	0.01	0.12	0.91

aCoef.: Multinomial regression coefficient adjusted for parent’s age group, relationship with the child, area of residence, highest education, and occupation. SE: Standard error.

## Data Availability

Data are not publicly available due to privacy or ethical restrictions.
